# Application of the electronic book to promote self-directed learning in medical technologist continuing education: a cross-sectional study

**DOI:** 10.1186/s12909-022-03724-w

**Published:** 2022-10-10

**Authors:** Chiao-Ni Wen, Chung-Guei Huang, Pi-Yueh Chang, Tsung-Han Yang, Huey-Ling You, Hsiao-Chen Ning, Kuo-Chien Tsao

**Affiliations:** 1 Department of Laboratory Medicine, Linkou Chang Gung Memorial Hospital, Taoyuan, Taiwan; 2 Department of Medical Biotechnology and Laboratory Science, Chang Gung University, Taoyuan, Taiwan; 3 Department of Medical Laboratories Administrative Center, Chang Gung Medical Foundation, Taoyuan, Taiwan; 4 Department of Laboratory Medicine, Kaohsiung Chang Gung Memorial Hospital, Kaohsiung, Taiwan; 5 Department of Medical Research and Development Linko Branch, Chang Gung Medical Foundation, Taoyuan City, 333 Taiwan, R.O.C.

**Keywords:** Continuing education, Electronic book, Self-directed learning, Continuing professional development, Medical technologist

## Abstract

**Background:**

Continuing education (CE) is essential for health professionals to improve competence in clinical practice, yet many medical technologists still experience barriers to learning in complex clinical settings. To better manage CE and address medical technologists’ learning needs, we developed a learner-centred electronic book (e-book) to promote self-directed learning for medical technologists.

**Methods:**

A cross-sectional study was conducted to explore the acceptability and learning impacts of the e-book as CE material for medical technologists in two medical centres in Taiwan. We designed the learner-centred context in the e-book based on medical technologists’ practice requirements and learning needs. Moreover, we adopted The New World Kirkpatrick Model with four levels (reactions, learning, behaviours and results) to evaluate the e-book’s learning impacts on medical technologists. A total of 280 medical technologists were invited to complete a questionnaire and a post-test, providing learning patterns as well as their satisfaction with the e-book and their learning outcomes after using it.

**Results:**

Most readers had positive learning experiences and better learning outcomes, including knowledge acquisition and behavioural change, after reading the e-book. The e-book became a new CE activity and reached medical technologists in various types of laboratories.

**Conclusions:**

The low-cost and learner-centred e-book effectively overcame CE learning barriers for medical technologists. The interactivity and flexibility of e-learning particularly helped learners to engage in clinical scenarios in laboratory medicine. This study could pave the way for medical educators to build a high-quality e-learning model in CE.

**Supplementary Information:**

The online version contains supplementary material available at 10.1186/s12909-022-03724-w.

## Background

Continuing education (CE) is fundamental for healthcare professionals to improve their competence and expertise in an ever-changing healthcare environment [1, 2]. CE is also essential for continuing professional development (CPD), which can drive individual and system changes to contribute to healthcare [3, 4]. While healthcare educators often provide comprehensive training programmes, many professionals still experience learning barriers in complex clinical settings [5, 6], such as feeling overwhelmed by heavy workloads [5–7], adapting themselves to different teaching styles and receiving learning materials irrelevant to their work [5, 6, 8]. To better manage CPD, professionals need to take responsibility for CE requirements throughout their professional careers [4, 9], and educators should investigate appropriate educational strategies to facilitate learners’ lifelong learning [10, 11].

Several studies [12–14] have shown that an effective educational programme could promote CE learning effectiveness using the following key methods: creating engagement, capturing attention, encouraging self-improvement and targeting learning needs. In spite of ample evidence of learning facilitators in CE, traditional educational meetings or teaching conferences, which are deemed to be ineffective and counterproductive, are still the most widely utilised programmes offered to healthcare professionals [15–17]. When educators or systems highly rely on lecture-based learning to share information, many professionals face learning barriers including passive learning [6, 8, 17], fixed schedules [6, 7, 16], short attention span [6, 7, 16], cognitive overload [7, 15, 17] and information redundancy [5, 6, 16]. However, due to a lack of effective educational programmes, many educators or systems still mainly hold educational conferences to meet CE requirements.

Over the past 20 years, self-directed learning (SDL) has been proven to help professionals to overcome learning barriers and acquire new skills and knowledge [17, 18]. SDL is widely used and includes eight elements: (a) it is a process (b) that is initiated by the individual, (c) which may or may not involve the help of others, (d) to identify their learning needs, (e) develop learning goals from these needs, (f) find the necessary resources to attain these goals, (g) select and implement the proper learning strategies to meet their goals, and (h) determine how to measure learning outcomes [19]. Most importantly, SDL encourages professionals to nurture self-motivation in their learning after work [8, 17, 18]. That is because the direct relevance of clinical needs and the consolidation of job knowledge are crucial to professionals, which could enhance their self-motivation towards regular learning [4, 19]. In addition, evidence-based research has shown that digital learning promotes SDL and that it is convenient for professionals to access courses through digital devices in clinical settings, positively impacting learners’ achievements [20, 21]. When digital media are designed to be emerging and habit-forming in the digital age, it is ideal for educators to use these technologies to enrich, extend, and advance learning in different environments [22, 23]. As a result, if educators could build a digital CE programme from the learner’s perspective, making the CE activity more intuitive, readable and enjoyable, they could effectively motivate professionals’ independent learning in various complex work environments.

According to recent investigations into digital learning interventions, electronic books (e-books) can successfully increase learners’ learning motivations through their page fidelity [24, 25], convenience [24–27] and interactivity [24–27] when compared to traditional learning approaches [24, 26]. The reason is that e-books, containing a combination of text, images and videos, including animations, self-test questions and other interactive activities, can be read and stored on mobile devices [25, 26]. Moreover, by interacting with the e-book content, learners significantly increase their learning motivation and metacognitive abilities to improve their learning achievement [24–26]. As a result, given its flexibility, accessibility, interactivity, usefulness, enjoyment potential and extensibility, the multimedia e-book could be an effective learning tool for professionals in complex clinical settings.

We aimed to optimise medical technologists’ learning experiences and address their learning needs by building a technology-enhanced CE programme. More importantly, two research questions were examined in this study: (1) Does the introduction of an enriched e-book incorporating interactive multimedia elements and learner-centred content could overcome learning barriers for medical technologists? and (2) What concerns do faculty members need to address to close learning gaps in CE for medical technologists? The objectives to achieve the research aim were as follows: 1) to develop an e-book to address learning barriers associated with traditional CE and promote self-learning, 2) to examine if the utilisation of the e-book overcomes learning barriers associated with traditional CE and 3) to evaluate the impact of the e-book on readers according to The New World Kirkpatrick Model, an outcome evaluation model of continuing medical education incorporating four domains or levels: reactions, learning, behaviours and results [28, 29].

## Methods

### Research design

We conducted a cross-sectional study from January to December 2018 to explore the acceptability and feasibility of using an e-book as a CPD programme for medical technologists in Taiwan. We designed the e-book as a quarterly book four times a year and published it in January, April, July, and October in 2018. After new releases in the e-book, we used convenient sampling to gather information from readers and medical technologists that could access the e-book through links as a CE program. They were informed that all respondents who achieved a score of at least 80% on the post-test would receive 1 h of CE credit. Once readers finished reading the e-book, they were invited to complete an online anonymous structured questionnaire and a post-test. To ensure anonymity and personal privacy, we did not collect any personally identifiable information. This study was approved by the Institutional Review Board of Chang Gung Medical Foundation, Taipei, Taiwan (IRB no. 201800144B0). The Chang Gung Medical Foundation Institutional Review Board approved to waive the documentation of informed consent.

### Study population

This study was conducted at the Department of Laboratory Medicine of two academic medical centres in Taiwan, specifically, Linkou Chang Gung Memorial Hospital and Kaohsiung Chang Gung Memorial Hospital, which employed 190 and 90 medical technologists, respectively. The inclusion criteria were full-time medical technologists worked in Linkou Chang Gung Memorial Hospital or Kaohsiung Chang Gung Memorial Hospital. The exclusion criteria were individuals employed as interns. All 280 medical technologists received e-mails or links to access different issues of the e-book quarterly. Study participants were defined as readers who completed the anonymous feedback questionnaire and post-test.

### Data collection

After new releases in the e-book, the questionnaire and post-test were made available online for 3 months. For example, after the e-book was issued in January 2018, data from the feedback questionnaire and the post-test completed between January and March 31 were collected and regarded as reader feedback and learning effects about the January 2018 e-book. The readers were also limited to submit data only once within the specific period. Data about the e-book were collected at four different periods.

### e-book design and learning context

**Fig. 1:**
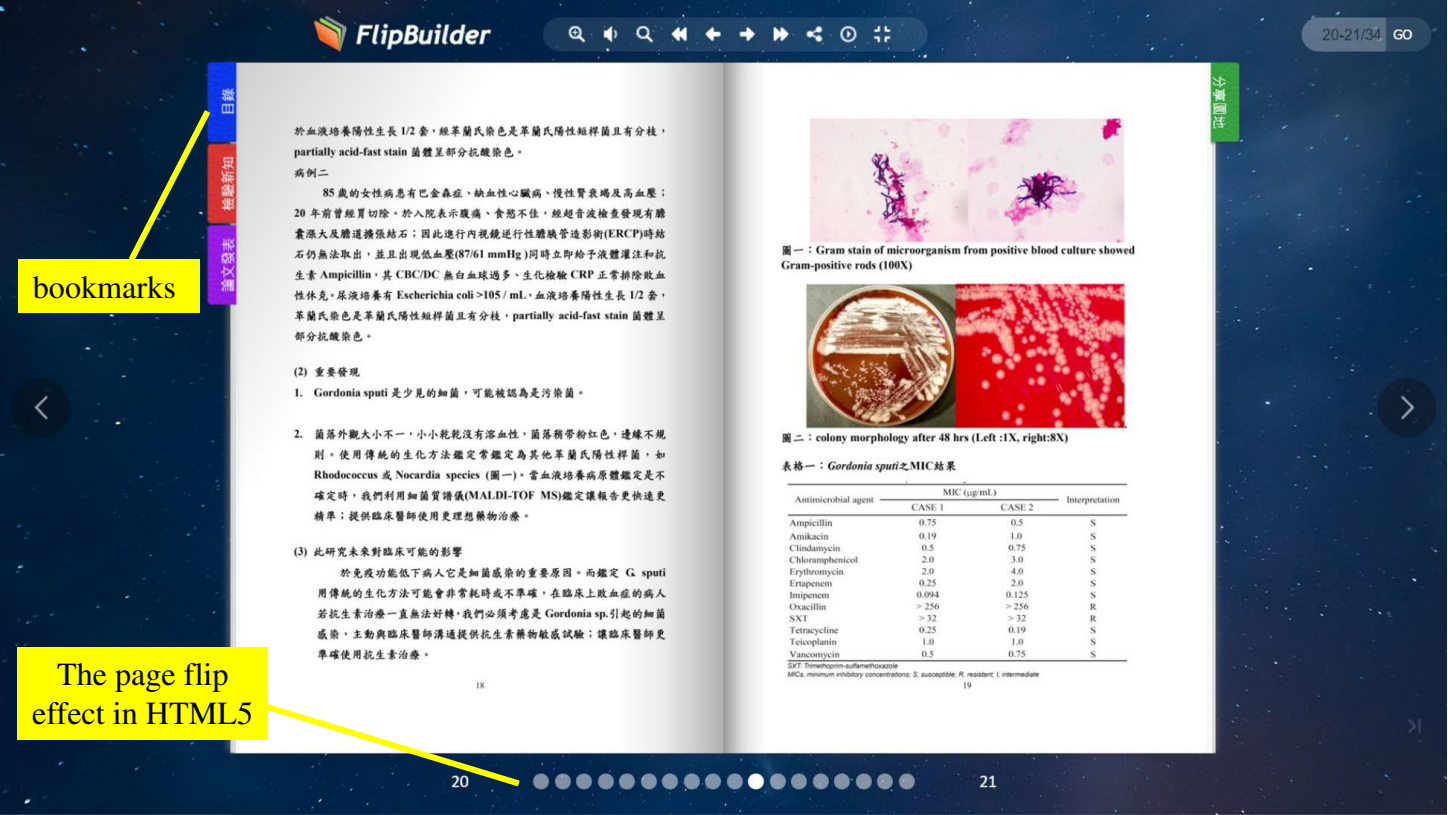
Advantages of e-book format for readers. The e-book format allows readers to download material from a website, view it online or easily email and read it offline

The learner-centred context was designed in an e-book format based on investigations on medical education and curricular development [3, 11] and medical technologists’ suggestions by using Flip PDF software version 4.1.10 (FlipBuilder, Hong Kong, China, https://www.flipbuilder.com; Fig. 1). With this e-book design software, articles and figures were resized to the final full text and PDF versions and then converted to HTML5. Additionally, the e-book editor could present dynamic content by adding videos, audio and links in the e-book. Readers could simultaneously access learning materials through the extension function provided by hyperlinks with the integration of multimedia information in the e-book. In addition, articles on knowledge application, information technology in the laboratory, quality improvement, clinical consulting, clinical research, and problem-solving examples were gathered, condensed, rewrote, and applied to the learning context. These learning topics were designed to meet the continuing competence programme requirements of Medical Technologists in Taiwan. In particular, since case-based learning is beneficial to learners’ engagement in specific clinical scenarios in laboratory medicine [30, 31], examples with pictures of the pre-analytical, analytical, and post-analytical characteristics were provided using e-modules to create a visualisation-based and interactive clinical case for learners. All components of the e-book were available in traditional Chinese. The content was approachable and relevant to clinical practice and had the ideal length. Most importantly, before each e-book was issued, the draft was reviewed by the editorial board of our department comprising senior medical technologists and experts in laboratory medicine.

### Outcome measurements

**Fig. 2:**
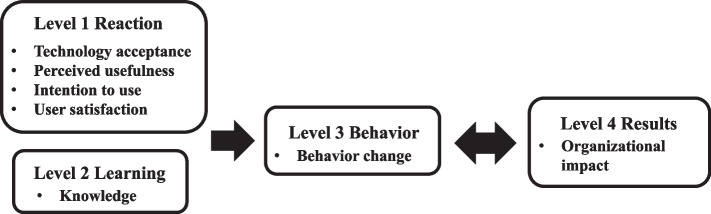
Adoption of The New World Kirkpatrick Model for evaluations of e-book on CE (modified with permission from [28]). This formal evaluation of CPD facilitated a more meaningful application of the e-book

Evaluation outcomes were adopted and analysed according to The New World Kirkpatrick Model [28], which incorporates four domains or levels: reactions, learning, behaviours, and results (Fig. 2). An anonymous structured questionnaire and a post-test with 10 multiple-choice questions about the corresponding knowledge were designed to assess readers’ perceptions, acceptance, and satisfaction towards the e-book (Level 1: reactions) and acquisition of knowledge (Level 2: learning). The number of readers who engaged with the e-book quarterly was used to measure the change in behaviour (Level 3: behaviours) since no e-book was published before the programme. Organisational changes (Level 4: results) were evaluated using the completion rate of 1 h of CE credit after they read the e-book since this CE credit was newly available in the organisation. Moreover, questions about learning styles and demographic characteristics were included in the survey to analyse the ability of the e-book to overcome learning barriers associated with traditional CE.

Readers were invited to respond immediately through hyperlinks placed at the end of the e-book. The feedback questionnaire was designed by the editorial board and reviewed by experts in laboratory medicine education. A pilot study of 10 individuals was conducted in January 2018 to pre-test the survey questions to determine how understandable these questions were. These questions were developed on the basis of a pilot study and experts’ opinions. The same feedback questionnaire was provided for readers of each issue to identify the changes in reading behaviours and the suggestion for future CE programs. Through the questionnaire, participants provided demographic information, learning patterns, assessments of the feasibility of using the e-book and their acceptance of satisfaction with using it, and learning outcomes after using it. Seven questions were included in the satisfaction measurements and rated on a 5-point Likert scale; an open-ended question was also included to express the respondents’ views on any topic of the e-book. A pilot study of 10 individuals was conducted to pre-test the survey questions in advance. Most people spent about 5 min to finish a 10-question survey, which was acceptable for most people. Furthermore, the questions in the post-test were designed by the editorial board and reviewed by experts in laboratory medicine education. The pre-test was particularly planned for each issue of the e-book, and the questions were designed to ask about the educational content in the e-book. Although each round had different questions, they were tested by the editorial board in advance to ensure that the level of difficulty of these questions was the same in each round for most medical technologists.

### Statistical analysis

Data were statistically analysed using Microsoft Excel and SAS software version 9.4 (SAS Institute, Cary, NC, USA). For categorical data, results were presented in tables by using frequencies or percentages. The Cochran–Armitage trend test was performed to determine the trends in binomial proportions across the levels of ordinal variables, whilst a chi-square test was performed to determine relationships between categorical variables. The phi correlation coefficient (phi) was used to measure the strength of the association between two categorical variables. Descriptive statistics were conducted to analyse scores and responses to Likert-type questions, which were presented as proportions (%), means, and standard deviations (SDs). In all analyses, two-tailed *p* < 0.05 was considered statistically significant.

## Results

A total of 280 medical technologists were invited to the study to complete the feedback questionnaire and the post-test during the study period in each round. The response rates to different issues of the e-book in 2018 were 25.0% (70/280), 43.9% (123/280), 43.6% (122/280), and 40.0% (112/280). The readers’ demographic characteristics, CE learning styles, and learning barriers were analysed. The demographic data of readers of different issues of the e-book had no significant differences (Table S1). Most of the study participants were women with a wide range of work experience. The most frequent laboratory specialty of all study participants was clinical chemistry, followed by clinical haematology and microscopy. Moreover, the largest percentages of readers receiving CE highly relied on hospital or healthcare delivery systems and professional associations, whereas more than 50% of the participants spent less than 30 min on educational activities per week. Overall, the major barriers faced by medical technologists in practicing CPD were lack of free time and information overload (Table S1).

Most respondents ‘agreed’ or ‘strongly agreed’ with the items measuring reader satisfaction on different issues of the e-book, including programme organisation, delivery modes, and learning stimulation (Table 1). To some extent, although readers reported less satisfaction in the first issue, satisfaction data in each round displayed the same pattern. The average of the responses to all seven items in each round was > 4, and the highest mean score was for the statement ‘The content and layout of the e-book stimulate my learning’. Moreover, the mean scores for the statement ‘Overall, I am satisfied with the e-book’ were higher than the average. This result indicated that the educational content in the e-book provided a favourable condition for CE. In the feedback, some respondents commented that they enjoyed the e-book for its interactivity (especially for clinical case scenarios), convenience, and topics highly related to clinical practice. However, a few respondents commented that ‘the e-book should focus on general specialty-related subjects’.

**Table 1:** Participant responses to the e-book from the satisfaction survey

Questionnaire Items	*Mean (SD)			
	1st issue (*n* = 70)	2nd issue (*n* = 123)	3rd issue (*n* = 122)	4th issue (*n* = 112)
I am satisfied with the contents and layout of the e-book	4.01 (0.86)	4.10 (0.81)	4.14 (0.70)	4.08 (0.63)
The contents and layout of the e-book stimulate my learning	4.13 (0.90)	4.15 (0.78)	4.18 (0.63)	4.12 (0.61)
It is convenient and attractive to use 3C to read the e-book	4.01 (0.89)	4.14 (0.79)	4.17 (0.69)	4.12 (0.64)
I am satisfied with the contents and layout of the journal articles in the e-book	4.03 (0.85)	4.09 (0.75)	4.15 (0.64)	4.07 (0.58)
The contents and layout of the journal articles in the e-book stimulate my learning	4.01 (0.86)	4.06 (0.75)	4.16 (0.66)	4.10 (0.58)
It is convenient and attractive to use 3C to read the journal articles in the e-book	4.01 (0.90)	4.11 (0.79)	4.20 (0.69)	4.13 (0.63)
Overall, I am satisfied with the e-book	4.06 (0.83)	4.15 (0.76)	4.18 (0.66)	4.13 (0.61)

^*^Responses based on a 5-point Likert scale (1 = strongly disagree, 5 = strongly agree)

**Fig. 3:**
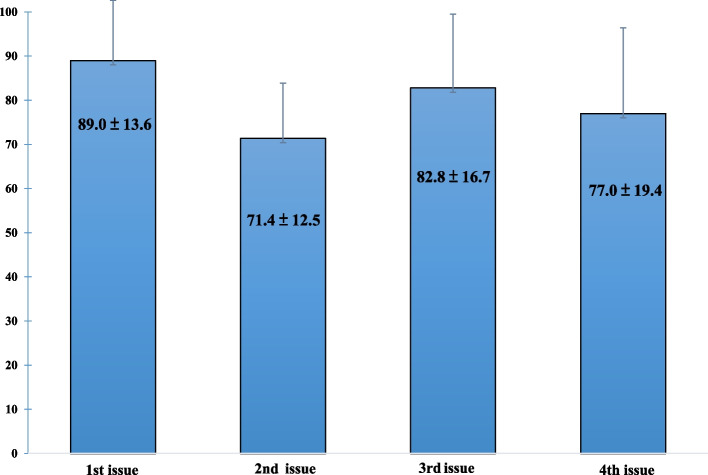
Study participants’ post-test results after they read different issues of the e-book

In the evaluation of learning effects on knowledge acquisition, readers in each round achieved high scores on the post-test (89.0%, 71.4%, 82.8%, and 77.0%, respectively) after they read different issues of the e-book (Fig. 3). Importantly, most participants mainly took courses provided by healthcare delivery systems (Table S1), whilst more than 50% of participants spent less than 30 min on educational activities per week. The data suggested that the reading engagement of the participants in this e-book programme was higher than that of the participants in previous CPD activities. Throughout the 1-year period, more than 70% of the readers achieved a score of at least 80% on the post-test in each round, successfully earning 1 h of CE credit since the e-book became a new CE activity at the Department of Laboratory Medicine.

In a further analysis of the associations between CPD participation frequency and readers’ self-perceived barriers, significant negative trends were found between participation frequency and language barriers (*p* trend = 0.0383) from the readers of the 2nd issue e-book (Table 2). Although the negative trends between participation frequency and language barriers were not significantly identified in the readers of other rounds, the frequency distribution indicated that readers with language barriers were more likely to spend less time on CPD programmes. Conversely, higher proportions of readers thought that the learning facilitators of the e-book, including the efficiency of conducting research, availability of the traditional Chinese language, optimal content length, and relevance to clinical practice, could help them overcome the corresponding learning barriers (Table 3). The strength of the relationship between readers’ self-perceived barriers and the corresponding learning facilitators of the e-book was measured using the phi correlation coefficient. Except for the facilitator ‘material-appropriate difficulty’, most readers with self-perceived barriers were more likely to report benefits from the corresponding facilitators including ‘efficiency for researching’, ‘available in Chinese’, ‘optimal content length’ and ‘relevance to clinical practice’. The positive correlation between identified barriers and corresponding facilitators could be detected in readers of different rounds although the correlation relationship did not show up in each round. Notably, the facilitator ‘available in Chinese’ was particularly beneficial to about 20 ~ 30% of readers with ‘language barriers’ in each round. (Table 3).

**Table 2:** Associations between study participants’ frequency of practicing CE and self-perceived barriers to CPD

Perceived barriers Readers of different e-books	Frequency of practicing continuing education per week (min) N (%)				
Lack of free time	< 15	15–30	31–60	> 60	*p* trend
1st issue (*n* = 70)	5 (15.2)	13 (39.4)	13 (39.4)	2 (6.1)	0.5593
2nd issue (*n* = 123)	13 (22.0)	26 (44.1)	13 (22.0)	7 (11.9)	0.7509
3rd issue (*n* = 122)	14 (21.9)	29 (45.3)	16 (25.0)	5 (7.8)	0.4068
4th issue (*n* = 112)	5 (10.2)	25 (51.0)	17 (34.7)	2 (4.1)	0.8809
Courses only offered in English					
1st issue	7 (25.0)	10 (35.7)	9 (32.1)	2 (7.1)	0.4965
2nd issue	15 (31.9)	20 (42.6)	8 (17.0)	4 (8.5)	**0.0383**
3rd issue	13 (25.0)	25 (48.1)	9 (17.3)	5 (9.6)	0.1812
4th issue	7 (16.7)	22 (52.4)	11 (26.2)	2 (4.8)	0.2900
Course length is too long to read					
1st issue	8 (17.4)	17 (37.0)	18 (39.1)	3 (6.5)	0.6397
2nd issue	14 (6.0)	33 (49.3)	18 (26.9)	12 (17.9)	0.1883
3rd issue	13 (15.9)	35 (42.7)	25 (30.5)	9 (11.0)	0.1174
4th issue	7 (10.3)	37 (54.4)	18 (26.5)	6 (8.8)	0.7202
Course content is too complicated to read					
1st issue	6 (25.0)	6 (25.0)	10 (41.7)	2 (8.3)	0.8150
2nd issue	6 (15.8)	18 (47.4)	12 (31.6)	2 (5.3)	0.9708
3rd issue	7 (16.7)	19 (45.2)	12 (28.6)	4 (9.5)	0.7178
4th issue	4 (8.7)	24 (52.2)	14 (30.4)	4 (8.7)	0.4614
Courses not related to clinical fields					
1st issue	5 (17.2)	9 (31.0)	14 (48.3)	1 (3.4)	0.5169
2nd issue	12 (20.3)	23 (39.0)	19 (32.2)	5 (8.5)	0.8390
3rd issue	8 (17.4)	18 (39.1)	12 (26.1)	8 (17.4)	0.1565
4th issue	6 (15.0)	18 (45.0)	12 (30.0)	4 (10.0)	0.7544

**Table 3:** Correlations between study participants’ perceived facilitators of e-book and self-perceived barriers to CPD

The phi correlation coefficient (95% CI) Perceived facilitators of CPD courses in e-books					
Perceived barriers	Save time for researching	Available in Chinese	Optimal-length articles	Material-appropriate difficulty	Related to clinical fields
Readers of different e-books					
Lack of free time					
1st issue	0.047 (-0.186 ~ 0.281)	**0.297 (0.074 ~ 0.520)**	-0.031 (-0.266 ~ 0.202)	-0.171 (-0.407 ~ 0.063)	-0.143 (-0.374 ~ 0.088)
2nd issue	**0.305 (0.134 ~ 0.475)**	0.005 (-0.171 ~ 0.181)	-0.035 (-0.212 ~ 0.140)	0.020 (-0.156 ~ 0.197)	-0.013 (-0.190 ~ 0.162)
3rd issue	**0.328 (0.148 ~ 0.507)**	0.038 (-0.139 ~ 0.215)	0.009 (-0.165 ~ 0.187)	**-0.287 (-0.480 ~ -0.095)**	-0.022 (-0.199 ~ 0.155)
4th issue	0.005 (-0.180 ~ 0.190)	0.131 (-0.050 ~ 0.317)	0.056 (-0.129 ~ 0.242)	0.011 (-0.174 ~ 0.197)	-0.007 (-0.192 ~ 0.177)
Courses only offered in English					
1st issue	-0.001 (-0.234 ~ 0.234)	**0.294 (0.073 ~ 0.516)**	0.046 (-0.187 ~ 0.281)	-0.192 (-0.416 ~ 0.031)	0 (-0.234 ~ 0.234)
2nd issue	**-0.183 (-0.365 ~ -0.001)**	**0.319 (0.157 ~ 0.481)**	-0.019 (-0.196 ~ 0.156)	-0.133 (-0.303 ~ 0.035)	-0.177 (-0.349 ~ -0.005)
3rd issue	0.099 (-0.075 ~ 0.273)	**0.225 (0.055 ~ 0.396)**	0.037 (-0.140 ~ 0.215)	-0.157 (-0.330 ~ 0.015)	-0.103 (-0.278 ~ 0.072)
4th issue	0.010 (-0.174 ~ 0.195)	**0.269 (0.096 ~ 0.442)**	0.153 (-0.034 ~ 0.342)	0.024 (-0.163 ~ 0.211)	-0.269 (-0.441 ~ -0.097)
Course length is too long to read					
1st issue	0.089 (-0.148 ~ 0.327)	-0.078 (-0.310 ~ 0.153)	0.199 (-0.029 ~ 0.429)	-0.018 (-0.255 ~ 0.218)	-0.120 (-0.353 ~ 0.112)
2nd issue	0.159 (-0.022 ~ 0.341)	-0.060 (-0.235 ~ 0.114)	0.145 (-0.026 ~ 0.317)	-0.043 (-0.223 ~ 0.136)	0.163 (-0.009 ~ 0.336)
3rd issue	0.059 (-0.121 ~ 0.241)	0.033 (-0.145 ~ 0.211)	**0.251 (0.083 ~ 0.419)**	0.026 (-0.148 ~ 0.201)	0.050 (-0.125 ~ 0.226)
4th issue	0.008 (-0.176 ~ 0.194)	-0.141 (-0.322 ~ 0.038)	0.065 (-0.117 ~ 0.248)	0.136 (-0.041 ~ 0.314)	0.137 (-0.042 ~ 0.317)
Course content is too complicated to read					
1st issue	0.035 (-0.196 ~ 0.268)	0.017 (-0.216 ~ 0.251)	0.041 (-0.193 ~ 0.275)	0.266 (-0.018 ~ 0.552)	**0.240 (0.011 ~ 0.470)**
2nd issue	-0.035 (-0.214 ~ 0.142)	0.110 (-0.060 ~ 0.281)	-0.116 (-0.288 ~ 0.055)	0.159 (-0.037 ~ 0.355)	**0.199 (0.020 ~ 0.379)**
3rd issue	0.0006 (-0.176 ~ 0.178)	0.065 (-0.109 ~ 0.240)	**0.250 (0.070 ~ 0.431)**	0.191 (-0.010 ~ 0.393)	0.064 (-0.114 ~ 0.243)
4th issue	-0.027 (-0.214 ~ 0.158)	**0.205 (0.027 ~ 0.383)**	0.097 (-0.089 ~ 0.289)	0.179 (-0.019 ~ 0.378)	0.185 (-0.002 ~ 0.373)
Courses not related to clinical fields					
1st issue	-0.221 (-0.456 ~ 0.015)	0.200 (-0.026 ~ 0.428)	**-0.271 (-0.496 ~ -0.046)**	-0.044 (-0.275 ~ 0.186)	0.145 (-0.087 ~ 0.377)
2nd issue	0.094 (-0.081 ~ 0.270)	-0.063 (-0.240 ~ 0.113)	0.098 (-0.077 ~ 0.275)	-0.062 (-0.238 ~ 0.114)	0.084 (-0.091 ~ 0.260)
3rd issue	0.079 (-0.094 ~ 0.252)	-0.022 (-0.200 ~ 0.155)	-0.016 (-0.193 ~ 0.160)	0.158 (-0.035 ~ 0.351)	**0.183 (0.003 ~ 0.362)**
4th issue	0.030 (-0.153 ~ 0.213)	-0.027 (-0.213 ~ 0.158)	-0.050 (-0.233 ~ 0.133)	-0.055 (-0.235 ~ 0.124)	**0.348 (0.152 ~ 0.543)**

## Discussion

The study aimed to develop an e-book that can address learning barriers associated with traditional CE and promote self-learning among medical technologists. To the best of our knowledge, this is the first study to create a low-cost quality e-learning model for medical technologists. Because it was free to provide the e-book online, the authors' work and editing process were the only costs involved in the production of the e-book. Furthermore, we adopted The New World Kirkpatrick Model with four levels (reactions, learning, behaviours and results) to evaluate the e-book’s learning impacts on medical technologists. Our study found impacts of the e-book at all four levels, furthering existing research that only examined the lower levels [23, 24]. The e-book became a new CE activity and reached medical technologists in various types of laboratories.

A major finding of this study is that most readers had positive learning experiences and better learning outcomes, including knowledge acquisition and behavioural change, after reading the e-book. However, while most previous investigations have focused on whether e-learning is effective generally [32–34], our study further assessed whether the e-book could be used to achieve effective learning outcomes in laboratory medicine. Moreover, we discussed different learning facilitators of the e-book, addressing how this novel educational intervention could have different types of learning effects on participants. Our study results indicated that information overload was inversely proportional to CPD participation frequency. As presented in the materials section, we gathered and condensed the latest information into articles of proper length, making the content easy to accept and read. Most readers held very positive attitudes towards the articles in the e-book and achieved high scores for corresponding questions. Similarly, previous CPD research projects providing concise and accessible updates to healthcare professionals also obtained positive results from respondents [35–37]. Based on a literature review of educational interventions [38, 39], the ‘concise writing’ style fits the core concept of cognitive load theory, which recommends that educators present information concisely to minimise the cognitive load.

In line with previous studies [32–34], e-learning education can provide results in terms of Kirkpatrick’s Levels 1 to 3 equivalent to those of traditional CE. However, in comparison, because e-learning education enables health professionals under various circumstances to overcome access issues, e-learning can more effectively achieve organisational goals in clinical settings [40–42]. In our study, one of the important factors supporting positive changes in organisational impact was the fact that the e-book did not require participants to have a high level of information technology literacy. Moreover, the e-book was offered to participants for free, which likely increased the participants’ commitment to the programme and positively affected attrition. Importantly, similar to a study in Chile [42], our e-learning CE activity was carried out in a systematic way and, as part of the CE programme, achieved Kirkpatrick’s four levels of organisational change.

Furthermore, we also found language barriers to be significant obstacles preventing non-native English speakers from acquiring updated knowledge in CPD programmes. Since language barriers were found to be significantly inversely proportional to CPD participation frequency, almost one-third of readers felt that the e-book’s availability in Chinese strongly supported their learning. Language barriers to medical education are common for non-native speakers, including health professionals in Asian countries [43, 44], Oceania country [45] and several European countries [46–48]. In the abovementioned countries, language barriers significantly hinder healthcare professionals’ learning. As a result, in the short term, it is proper to provide language support to professionals struggling due to language barriers; in the long term, further studies and discussions are needed to find a better solution.

To our knowledge, there are no published studies regarding CE that facilitates e-learning while considering medical technologists’ views and preferences. We tried to create a high-quality but low-cost e-learning model to meet the strong demand from both educators and medical technologists. In addition to the abovementioned components, our evaluation also indicated that it is critical for CPD programmes to be useful to learners’ practice. The relative requirements of CE were also found in previous studies on different types of healthcare professionals [49, 50]. We suggest that CPD programmes focus on the actual and predicted practice requirements that could improve learners’ clinical skills and competence. We chose the e-book as the multimedia learning tool for CE for two reasons. First, from the learners’ perspective, the e-book is convenient to use and relevant to medical technologists’ life experiences, since the popularity of reading e-books using mobile devices has been growing rapidly in Taiwan. Second, from the educators’ perspective, it is cheap and easy to produce e-books using PDF files as the basic template for conversions. Moreover, e-books are a powerful educational tool to achieve highly interactive and active learning experiences in medical education. Furthermore, e-books are simple, and they can be disseminated in all clinical settings [23, 24].

Our study has some limitations. This was a cross-sectional study, and there was no control group. Thus, we could not measure the effectiveness of our e-learning model compared to traditional methods. Furthermore, we did not attempt to determine whether our model was more effective; this study aimed to build a low-cost e-learning model for medical technologists and explore how medical technologists perceived the e-book. In this study, the participants were defined as readers who completed the questionnaire and post-test. Therefore, the same medical technologist could have answered the questionnaire for different issues of the e-book and been viewed as different readers. Considering the observations from each round may not be independent from each other, we have presented the results of each round separately. According to the inconsistent results in each round, it seems reasonable to assume that there were demographic differences among readers in each round. Finally, although we identified and then used many facilitators to improve learners’ learning efficiency, it was still difficult to measure the true impact of different facilitators on learning. Because we did not identify and collect data on possible confounding factors, we could not control the influence of any covariates on learning. Such outcomes should be addressed in future studies.

## Conclusions

Our cross-sectional study demonstrated that our low-cost and learner-centred e-book effectively overcame many learning barriers in CE for medical technologists. The interactivity and flexibility of e-learning particularly helped learners to engage in clinical scenarios in laboratory medicine. Today, with the distinctive rise of e-learning, our study could pave the way for educators to build a high-quality e-learning model for professionals in CE.

## Supplementary Information

**Additional file 1:** **Table S1.** Demographic characteristics and CE learning styles of participants.

## Data Availability

The datasets used and analysed during the current study are available from the corresponding author on reasonable request.

## Abbreviations

CE: Continuing education
CPD: Continuing professional development
SDL: Self-directed learning
e-book: Electronic book
