# Supplementary material for: Application of the electronic book to promote self-directed learning in medical technologist continuing education: a cross-sectional study
Source: BMC Med Educ. 2022 Oct 10;22:713. doi: 10.1186/s12909-022-03724-w (PMC9549609; doi:10.1186/s12909-022-03724-w)
Supplement: Supplementary file 1 — Additional file 1: Table S1. Demographic characteristics and CE learning styles of participants. [file 12909_2022_3724_MOESM1_ESM.docx]

**Table S1**. Demographic characteristics and CE learning styles of participants.

| Characteristic/readers of different e-boos | 1st issue | 2nd issue | 3rd issue | 4th issue | *p* value |
| --- | --- | --- | --- | --- | --- |
|  | 70 | 123 | 122 | 112 |  |
| Gender, N (%) |  |  |  |  |  |
| Female | 53 (75.7) | 80 (67.5) | 81 (66.4) | 74 (66.1) | 0.5189 |
| Male | 17 (24.3) | 43 (32.5) | 41 (33.6) | 38 (33.9) |  |
| Work experience in laboratory medicine | | | | | |
| ≤ 5 | 6 (8.6) | 18 (14.6) | 23 (18.9) | 22 (19.6) | 0.6103 |
| 6-10 | 5 (7.1) | 15 (12.2) | 18 (14.8) | 13 (11.6) |  |
| 11-15 | 13 (18.6) | 17 (13.8) | 15 (12.3) | 18 (16.1) |  |
| 16-20 | 13 (18.6) | 26 (21.1) | 23 (18.9) | 24 (21.4) |  |
| 21-25 | 21 (30.0) | 25 (20.3) | 24 (19.7) | 17 (15.2) |  |
| ≥ 26 | 12 (17.1) | 22 (17.9) | 19 (15.6) | 18 (16.1) |  |
| Laboratory specialty (Please select all that apply) | | | | | |
| Clinical chemistry | 18 | 41 | 44 | 38 |  |
| Clinical haematology | 19 | 37 | 41 | 37 |  |
| Clinical microscopy | 14 | 33 | 38 | 30 |  |
| Leading source for CPD events (Please select all that apply) | | | | | |
| Hospital/Health care delivery system | 58 | 101 | 101 | 83 |  |
| Professional associations | 51 | 76 | 68 | 62 |  |
| Multimedia CPD | 13 | 43 | 35 | 33 |  |
| Industry-sponsored CPD | 30 | 66 | 58 | 51 |  |
| Journal-based CPD | 23 | 43 | 39 | 35 |  |
| Textbooks | 10 | 12 | 9 | 6 |  |
| Time for CPD per week (min) | | | | | |
| < 15 | 12 (17.1) | 24 (19.5) | 25 (20.5) | 16 (14.3) | 0.5209 |
| 15–30 | 28 (40.0) | 55 (44.7) | 53 (43.4) | 54 (48.2) |  |
| 31–60 | 27 (38.6) | 31 (25.2) | 31 (25.4) | 33 (29.5) |  |
| >60 | 3 (4.3) | 13 (10.6) | 13 (10.7) | 9 (8.0) |  |
| Major barriers to practice CPD (Please select all that apply) | | | | | |
| Lack of free time | 33 | 59 | 64 | 49 |  |
| Courses only offered in English | 28 | 47 | 52 | 42 |  |
| Course length is too long to read | 46 | 77 | 82 | 68 |  |
| Course content is too complicated to read | 24 | 38 | 42 | 46 |  |
| Courses not related to clinical fields | 29 | 59 | 46 | 40 |  |
